# Interpretation of 11C–choline PET/CT for the diagnosis of local relapse in radically treated prostate cancer

**DOI:** 10.1186/s41824-017-0007-x

**Published:** 2017-10-12

**Authors:** A. Matti, G. M. Lima, L. Zanoni, C. Pultrone, R. Schiavina, F. Lodi, S. Fanti, C. Nanni

**Affiliations:** 1 Nuclear Medicine Department, S.Orsola-Malpighi Hospital, Bologna, Italy; 2 Urology Department, S.Orsola-Malpighi Hospital, Bologna, Italy

**Keywords:** 11C–Choline PET/CT, Prostate cancer, Radical prostatectomy, Local relapse, Biochemical relapse

## Abstract

**Purpose:**

11C–choline PET/CT is a widely-used tool for the diagnostic of prostate cancer (PCa). In literature, a great variability of local relapse (LR) detection rate is reported. The aim of this study is to provide positivity criteria for 11C–choline PET/CT detection of LR in patients who had surgery for PCa and presented prostate specific antigen (PSA) failure.

**Methods:**

Sixty patients radically treated for PCa and presenting PSA failure were retrospectively analysed. Two Nuclear Medicine Physicians revised the 11C–choline PET/CT scans and defined by consensus if even mild focal uptake was present in the prostate bed (PB) and bladder-urethral junction (BUJ) along midline, regardless the previous report results.

The results were subsequently correlated with a clinical and radiological follow up (FU) of 1 to 2 year and with TNM staging, Gleason score (GS), PSA level at relapse, radiotherapy (RT) and hormone therapy (HT) after surgery.

**Results:**

There was focal uptake in 22/60 patients; 11 of them were true positive and 11 false positive. The PSA level showed a tight connection with the true positivity/negativity of Choline scan. Most of true positive cases (10/11 patients) presented a PSA ≥ 1 ng/ml, while approximately half of the false positive cases (5/11 patients) presented PSA below 1 ng/ml. The other variables were not correlated to Choline detection rate for LR.

**Conclusions:**

This study shows that an even mild focal uptake of Choline in the PB and BUJ along midline must be considered suspicious for LR in patients radically treated for PCa, especially if they are presenting with PSA level > 1 ng/ml.

## Main text

Prostate Cancer (PCa) is the most frequently diagnosed malignancy in men worldwide. Radical prostatectomy (RP) is considered as the gold standard for cancers confined to the prostate and lymph nodes. However, despite a radical approach, PCa relapse is relatively common occurring in approximately 35% of patients within 10 years after RP, and in 50% after external-beam radiotherapy (Han et al., 2003). The most powerful tool available to identify an early relapse is the detection of a progressively increasing level of serum PSA. After RP, a serum PSA level higher than 0,2 ng/mL, confirmed by two subsequent consecutive measures, is considered expression of either residual or recurrent disease (Mottet et al., 2011). In these cases, the exact localization of disease relapse is reached through a combination of various imaging procedures, such as trans-rectal ultrasound (TRUS), pelvic magnetic resonance (MR) and bone scan (BS). However their sensitivity is recognized to be suboptimal, especially for low PSA levels (Coakley et al., 2004; Choueiri et al., 2008).

^11^C–choline PET/CT is another widely-used functional imaging procedure for PCa detection (Fanti et al., 2016; Evangelista et al., 2013). Choline is an essential component of cell membrane. PCa presents an increased cell proliferation and up-regulation of choline kinase and choline transporters, as well as an increased expression of choline transporters, leading to an increased uptake of this tracer (Muller et al., 2009). In particular, several studies have shown that ^11^C–choline PET/CT detection rate of LR, lymph node involvement and bone metastases in patients with biochemical failure after RP is related to trigger PSA serum levels (Krause et al., 2008).

In literature (Evangelista et al., 2015; Reske et al., 2008), a great variability of LR detection rate is reported for Choline PET/CT and this is mostly due to the absence of standardized image interpretation criteria. This is true especially for findings located in the prostate bed (PB) and in the bladder-urethral junction (BUJ) along midline. Even though this findings occur in a minor number of cases, the possible presence of radioactive urine may lead to underrate or overestimate areas of focal uptake. The correct identification of sites of early recurrence may improve the capability of physician to predict the clinical outcome and to propose appropriate therapies.

The aim of this study is to analyze the performance of ^11^C–choline PET/CT for the assessment of local relapse and to provide positivity criteria for ^11^C–choline PET/CT detection of LR in patients radically treated for PCa and presenting with PSA failure and equivocal focal uptake in the PB and BUJ.

### Patient population

This study was performed according to the declaration of Helsinki and to national regulations. All the patients provided informed consent for participation and anonymous publication of data.

Sixty patients (56 to 79 years old; mean 67.5) radically treated for PCa (RP ± RT ± HT) and presenting with increased PSA serum level (0.2–16 ng/mL, mean 8.1 ng/mL) were retrospectively analysed. All the patients had undergone 11C–Choline PET/CT. At the time of PET/CT scans, 2 patients were receiving HT (but with rising PSA), while all the others were out of any PCa specific therapy.

### Radiopharmaceutical

^11^C–choline was synthesized according to the solid-phase method as described by Pascali et al. (Pascali et al., 2000) using a commercial synthesis module (TracerLab; GE Medical System, Waukesha, WI, USA).

### Imaging protocol

Patients were studied with ^11^C–choline PET/CT that was performed following standard procedure. Patients received an intravenous injection of 478.6 **±** 72.5 MBq of ^11^C–choline and scan started immediately after the injection. All the examinations were obtained with a hybrid PET/CT scanner (Discovery DSTE; GE Medical System, Waukesha, WI, USA).

### Image analysis and validation criteria

Two Nuclear Medicine Physicians, with more than 10 years of experience with choline imaging, independently revised and reinterpreted the 11C–choline PET/CT scans and categorized the results as positive or negative for local abnormal uptake in PB and BUJ along midline. Only focal uptake visually detected, above the surrounding background, was considered as abnormal. Reviewers were blind to PSA level at the moment of the scan. Equivocal cases (6/60), related to the intensity of the focal uptake, were discussed and final categorization was reached, thanks to a further scan revision performed by a third expert Nuclear Medicine Physician, with more than 10 years of experience with choline imaging.

The 11C–Choline PET/CT results were validated in the light of subsequent clinical and radiological follow up (FU) of 1 to 2 year, including PSA trend after targeted therapy (such as RT on the prostatic bed), other imaging modalities (such as pelvic MR and/or TRUS) and/or local biopsies.

### Analysis of results

Sixty patients radically treated for PCa and presenting with PSA failure were retrospectively enrolled and their ^11^C–choline PET/CT scans were reviewed, regardless the previous report. Overall, focal uptakes (even mild) were described in the PB and BUJ along midline in 22 out of 60 patients (36.7%). These results were subsequently correlated with a clinical and radiological follow up (FU) of 1 to 2 years, based on different diagnostic procedures that could either confirm or exclude the presence of LR (TRUS-guided biopsy, pelvic MR and/or PSA trend after targeted therapy).

The 11C–Choline PET/CT scans were considered positive for LR if the clinical and radiological FU reported alternately:

- MR or TRUS evidences of LR;
- positive histological test provided through local biopsies;
- normalization of the PSA serum level after targeted therapy.

**Fig. 1:**
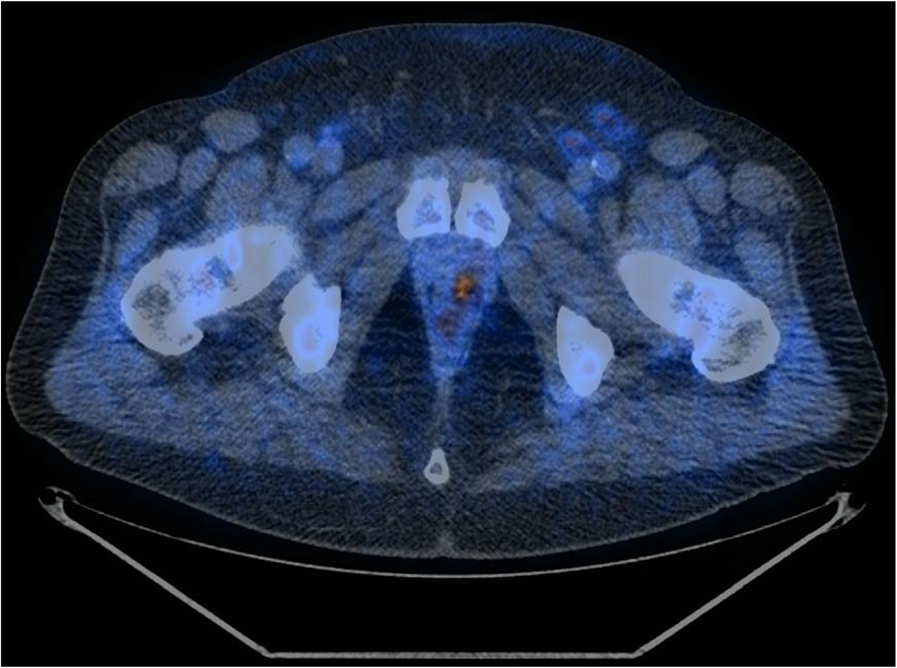
True positive case (PET/CT axial scan). This scan shows a focal uptake of 11C–choline localized on the left paramedian versant of the PB

**Fig. 2:**
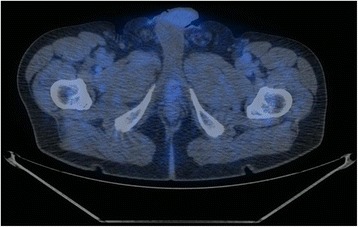
True positive case (PET/CT axial scan, PET only axial scan, MIP). This scan shows a focal uptake of 11C–choline localized in PB, right behind the bladder

**Fig. 3:**
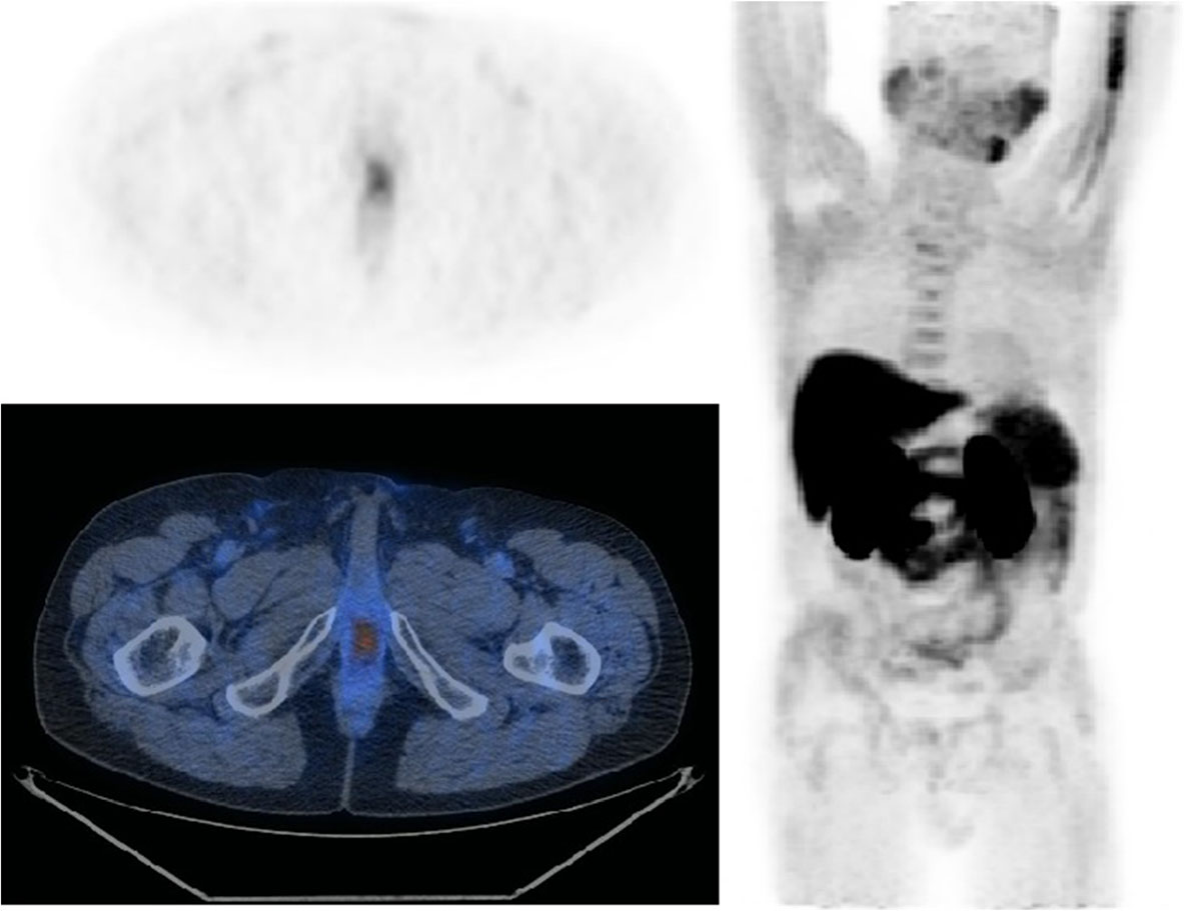
False positive case (PET/CT axial scan). This scan shows a mild focal uptake of 11C–choline localized in the BUJ along midline

**Fig. 4:**
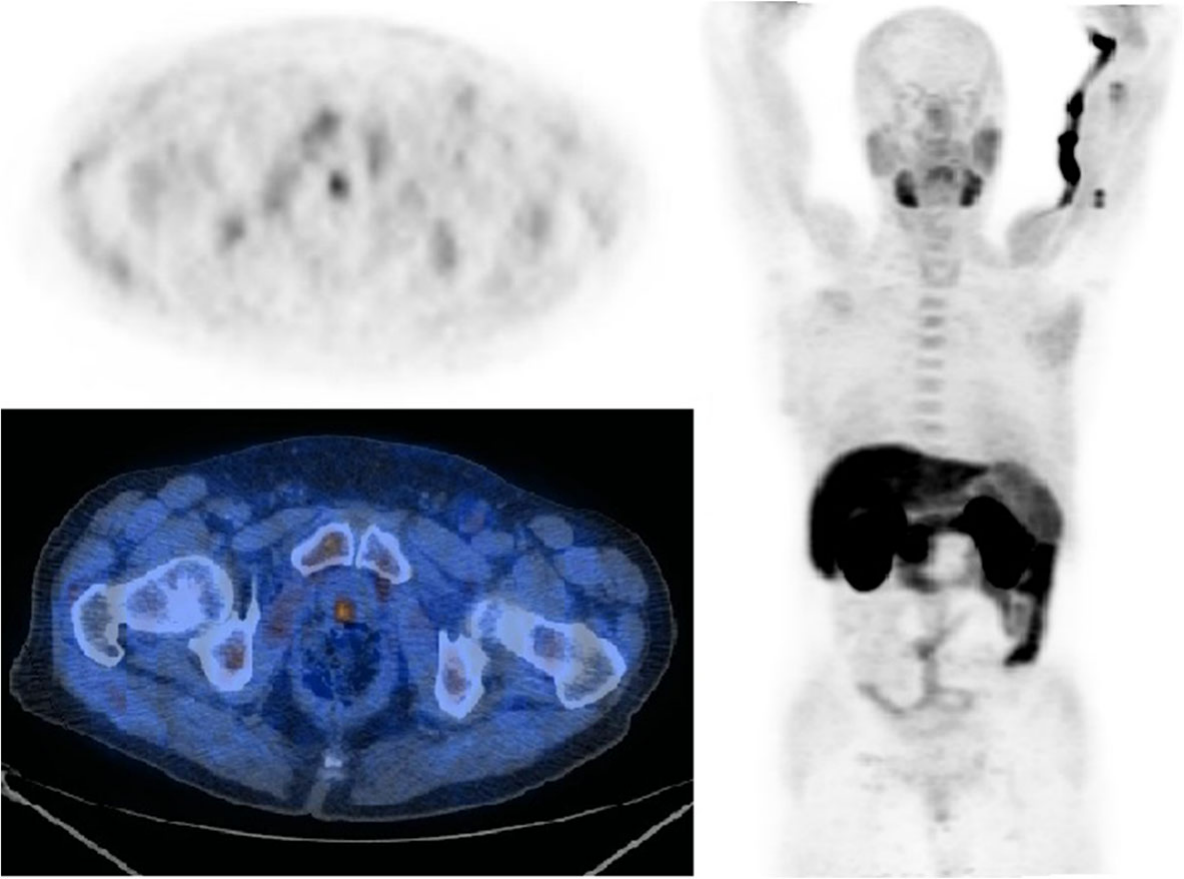
False positive case (PET/CT axial scan, PET only axial scan, MIP). This scan shows a focal uptake of 11C–choline localized in the BUJ along midline

Overall 22 scans were defined positive whereas the remaining 38 negative for LR. Eleven resulted true positive (18.3%, see Fig. 1 and Fig. 2), 11 false positive (18.3%, see Fig. 3 and Fig. 4), 4 false negative (6.7%) and 34 true negative (56.7%). In this particular setting, 11C–choline PET/CT presented a sensitivity of 73.3% and a specificity of 75.6% (see Table 1).

**Table 1:** Summary of patient distribution. In this table it is shown in detail the distribution of patients according to the four possible categories: true positive, false positive, true negative and false negative. On the right there sensitivity and specificity are presented

Summary Table				
Reported findings	22	11 True positive	18.3%	Sensitivity = TP/TP + FN = 73.3%
		11 False positive	18.3%	
Rest of the population	38	4 False negative	6.7%	Specificity = TN/TN + FP = 75.6%
		34 True Negative	56.7%	
Total	60		100%	

Furthermore, the comparison of the results of the re-analysis of the images with the original reports (12 true positive, 2 false negative, 30 true negative and 16 false positive) has shown a higher sensitivity (85%) but a significantly lower specificity (65%).

Therefore, these results were correlated to several possible predictive variables, such as TNM staging, GS, PSA serum level at the time of the execution of the exam, RT and HT after surgery. This assessment demonstrated that most of the true positive cases (10/11) presented a PSA serum level at the time of the scans ≥1 ng/mL, while nearly a half of the false positive cases (5/11) presented a lower serum level of PSA.

The other variables (TNM staging, Gleason score, RT and HT after surgery) showed no influences on LR detection rate.

## Discussion

In literature, a great variability detection rate is reported for ^11^C–choline PET/CT. Krause BJ et al. (Krause et al., 2008) reported 36% in patients presenting PSA serum level < 1 ng/mL after primary therapy for PCa, Nanni et al. (Nanni et al., 2013) reported 20% in radically treated patients and presenting PSA failure (>0.3 ng/mL), Giovacchini et al. (Giovacchini et al., 2010) reported 24% in patients radically treated and presenting PSA serum level < 1.4 ng/mL and 68% with PSA serum level > 1.4 ng/mL, Castellucci et al. (Castellucci et al., 2014) reported 28.4% in patients presenting early biochemical relapse (<2 ng/mL) after RP. This issue is relevant not for ^11^C–choline PET/CT only, but for almost all PET radiopharmaceuticals employed in prostate cancer imaging, including the more recent ^68^Ga-PSMA and ^18^F–FACBC. There is a strong need to define standardized image interpretation criteria, so that patients will benefit from a more accurate and personalized therapeutic planning.

In order to minimize these differences and to define new interpretation criteria, every focal uptake in the PB and BUJ along midline, that have been reported in this study, was correlated to TNM staging, GS, PSA serum level at the time of the execution of the exam, RT and HT after surgery, as possible predictive variables.

Despite the limitations of this study (low number of patients, possible presence of radioactive urine in the bladder and urethra, resolution limitation), it was possible to make some considerations.

**Fig. 6:**
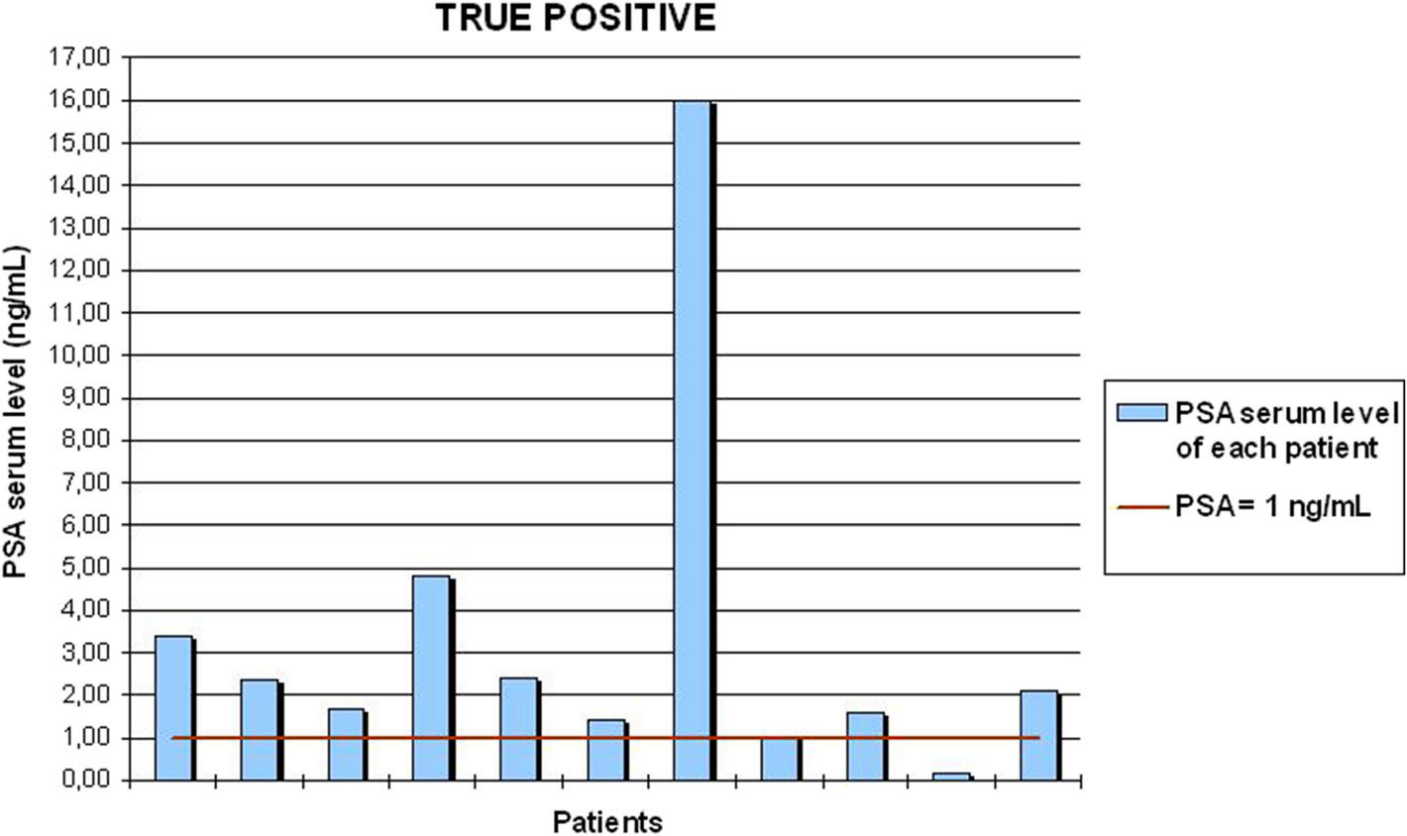
PSA serum level of the true positive cases. This graph shows in detail the trigger PSA of all the true positive cases described in this study, demonstrating that the majority of true positive cases (10/11) has a PSA serum level ≥ 1 ng/ml

This analysis showed a strong correlation between the serum PSA level at the time of the exam and the true positivity/negativity of ^11^C–choline PET/CT scans for LR. In fact, most of true positive cases (10 out of 11 patients) were associated with a PSA serum level ≥ 1 ng/mL, while approximately half of the false positive cases (5 out of 11 patients) presented a serum level of PSA below 1 ng/mL (see Fig. 5 and 6). According to our preliminary results, every focal uptake in the PB or BUJ along midline is likely to be a LR and should be reported. In addition, if the PSA serum level at the time of the scans is ≥1 ng/mL, this finding is highly specific (see Table 2).

**Fig. 7:**
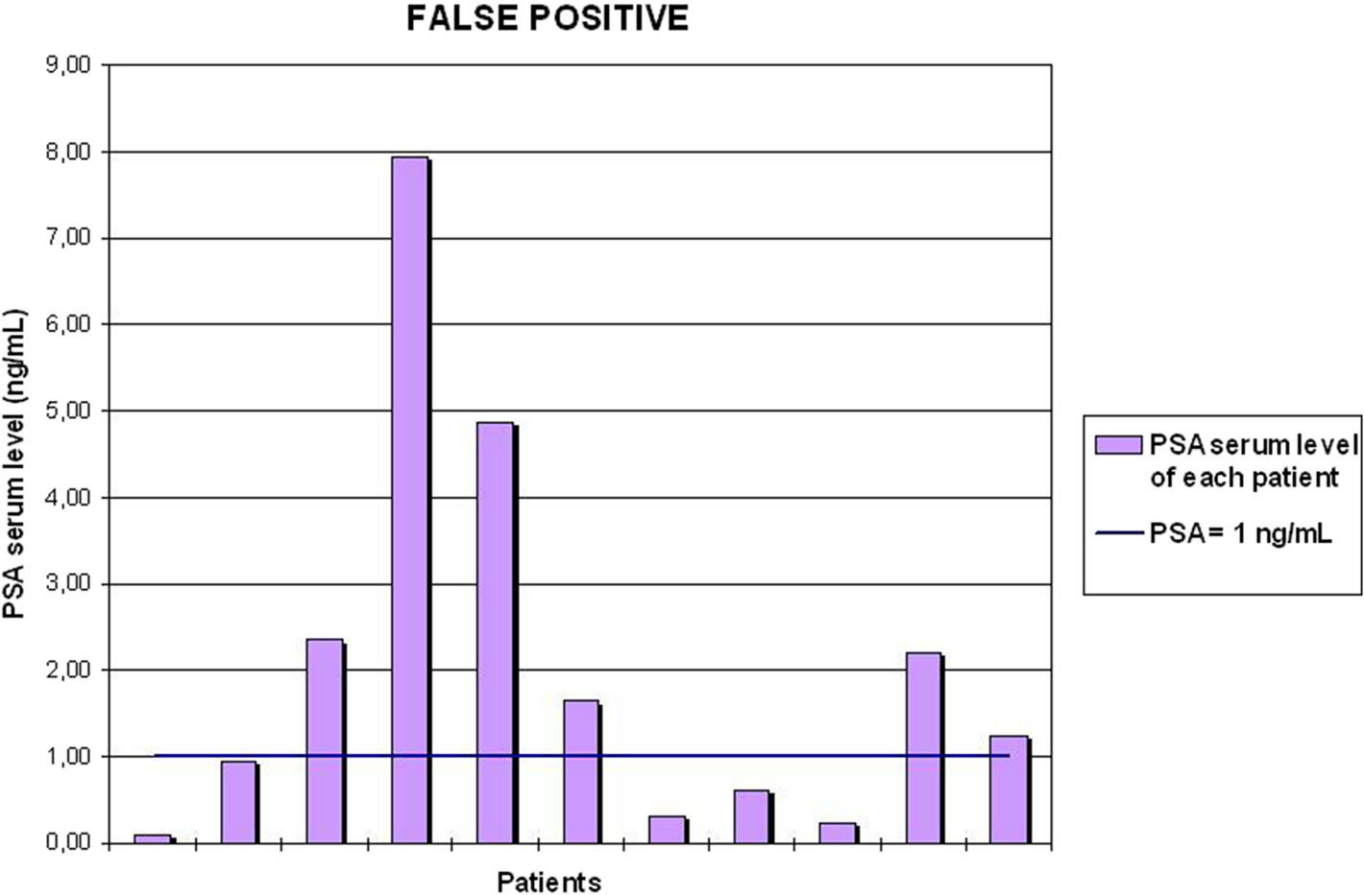
PSA serum level of the false positive cases. This graph shows in detail the trigger PSA of all the true positive cases described in this study, demonstrating that nearly a half of the false positive cases (5/11) has a PSA serum level < 1 ng/ml

**Table 2:** Summary of patient distribution, related to PSA. In this table it is shown in detail the distribution of patients, according to the four possible categories and related to the PSA serum level at the time of the scans. On the right there are reported the values of sensitivity and specificity of this analysis

Summary Table with PSA ≥ 1 ng/mL				
Reported findings	16	10 True positive	16.7%	Sensitivity = TP/TP + FN = 66.7%
		6 False positive	10%	
Rest of the population	44	5 False negative	8.3%	Specificity = TN/TN + FP = 86.7%
		39 True Negative	65%	
Total	60		100%	

## Conclusion

This study shows that an even mild focal uptake of Choline in the PB and BUJ along midline must be considered suspicious for LR and has to be reported, especially in patients radically treated for PCa and presenting with PSA serum level ≥ 1 ng/ml. Further studies including a larger number of patients prospectively enrolled are needed to confirm our preliminary findings.

## Abbreviations

BS: Bone scan
BUJ: Bladder-urethral junction
FU: Follow up
GS: Gleason Score
HT: Hormone therapy
LR: Local relapse
MR: Pelvic magnetic resonance
PB: Prostate bed
PCa: Prostate cancer
PSA: Prostate specific antigen
RP: Radical prostatectomy
RT: Radiotherapy
TRUS: Trans-rectal ultrasound
